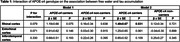# APOE4 moderates the association between cerebral small vessel disease and cortical tau accumulation in midlife

**DOI:** 10.1002/alz.091824

**Published:** 2025-01-09

**Authors:** Liliana Espinoza, Matthew R Scott, Alexa S Beiser, Andy Q Banh, Vinu Philip, Saptaparni Ghosh, Emma G Thibault, Pauline Maillard, Charles Decarli, Georges El Fakhri, Keith A Johnson, Sudha Seshadri, Claudia L Satizabal

**Affiliations:** ^1^ Glenn Biggs Institute for Alzheimer's & Neurodegenerative Diseases, University of Texas Health San Antonio, San Antonio, TX USA; ^2^ Boston University School of Public Health, Boston, MA USA; ^3^ The Framingham Heart Study, Framingham, MA USA; ^4^ Boston University Chobanian & Avedisian School of Medicine, Boston, MA USA; ^5^ Washington University School of Medicine, St. Louis, MO USA; ^6^ University of Texas Health San Antonio Long School of Medicine, San Antonio, TX USA; ^7^ Gordon Center for Medical Imaging, Massachusetts General Hospital, Boston, MA USA; ^8^ Alzheimer's Disease Research Center, University of California Davis, Sacramento, CA USA; ^9^ Gordon Center for Medical Imaging, Massachusetts General Hospital, Harvard Medical School, Boston, MA USA; ^10^ Glenn Biggs Institute for Alzheimer’s & Neurodegenerative Diseases, University of Texas Health Sciences Center at San Antonio, San Antonio, TX USA; ^11^ Boston University School of Medicine, Boston, MA USA

## Abstract

**Background:**

Recent research suggests that soluble pathogenic tau accumulates in the brain microvasculature of patients with Alzheimer’s Disease (AD) and primary tauopathies, driving cerebrovascular impairments and further tau accumulation. However, little is known about the interplay of these processes before dementia onset. In the present study, we investigated the association between free water (FW), an early biomarker of cerebral small vessel disease, and tau accumulation in the rhinal, entorhinal, and inferotemporal cortices derived from PET imaging in middle‐aged adults. Additionally, given the known increased risk of AD among APOE‐ɛ4 carriers, we further explored the interaction between FW and APOE‐ɛ4 carriership on tau accumulation.

**Method:**

We included 281 cognitively healthy participants (mean age 55±9 years, 49% male, 23% APOE‐ɛ4 carriers) from the Third Generation Cohort of the Framingham Heart Study who had undergone brain MRI and tau PET imaging. We used linear regression models to relate FW to regional tau, adjusting for potential confounders. Model 1 was adjusted for age, age^2^, sex, camera, and time interval between MRI and PET scans. Model 2 was additionally adjusted for cardiovascular risk factors (i.e. systolic blood pressure, antihypertensive medication use, type 2 diabetes, body mass index, smoking, and prevalent cardiovascular disease).

**Result:**

Although we did not find a significant association between FW and regional tau, we observed significant interactions between FW and APOE‐ ɛ4 genotype on tau accumulation in the rhinal (p=0.079) and entorhinal cortices (p=0.071) in fully adjusted models (Table 1). Among APOE‐ɛ4 carriers, we found a significant association between higher FW and increased rhinal tau levels (β=1.49, p=0.031), and a borderline significant association between higher FW and increased entorhinal tau (β=1.25, p=0.061). No significant associations were observed in non‐APOE‐ɛ4 carriers.

**Conclusion:**

Our findings suggest that the APOE‐ɛ4 genotype is a moderator in the association between cerebrovascular injury and tau accumulation in brain regions typically affected earlier in the pathological spreading of tau in AD. These findings may help further elucidate underlying early processes leading to tau propagation. Replication studies in larger samples are needed to confirm these findings.